# Preliminary Evidence for Cell Membrane Amelioration in Children with Cystic Fibrosis by 5-MTHF and Vitamin B12 Supplementation: A Single Arm Trial

**DOI:** 10.1371/journal.pone.0004782

**Published:** 2009-03-11

**Authors:** Cinzia Scambi, Lucia De Franceschi, Patrizia Guarini, Fabio Poli, Angela Siciliano, Patrizia Pattini, Andrea Biondani, Valentina La Verde, Oscar Bortolami, Francesco Turrini, Franco Carta, Ciro D'Orazio, Baroukh M. Assael, Giovanni Faccini, Lisa M. Bambara

**Affiliations:** 1 Department of Clinical and Experimental Medicine, Section of Rheumatology & Internal Medicine, University of Verona, Verona, Italy; 2 Department of Medicine and Public Health, Unit of Epidemiology and Medical Statistics, University of Verona, Verona, Italy; 3 Department of Genetic, Biological and Medical Chemistry, Section of Medical Chemistry, University of Torino, Torino, Italy; 4 Nurex, Sassari, Italy; 5 Center for Cystic Fibrosis Care, Hospital of Verona, Verona, Italy; 6 Department of Biomedical and Morphological Science, Section of Clinical Chemistry, University of Verona, Verona, Italy; Max F. Perutz Laboratories, Austria

## Abstract

**Background:**

Cystic fibrosis (CF) is one of the most common fatal autosomal recessive disorders in the Caucasian population caused by mutations of gene for the cystic fibrosis transmembrane conductance regulator (CFTR). New experimental therapeutic strategies for CF propose a diet supplementation to affect the plasma membrane fluidity and to modulate amplified inflammatory response. The objective of this study was to evaluate the efficacy of 5-methyltetrahydrofolate (5-MTHF) and vitamin B12 supplementation for ameliorating cell plasma membrane features in pediatric patients with cystic fibrosis.

**Methodology and Principal Findings:**

A single arm trial was conducted from April 2004 to March 2006 in an Italian CF care centre. 31 children with CF aged from 3 to 8 years old were enrolled. Exclusion criteria were diabetes, chronic infections of the airways and regular antibiotics intake. Children with CF were supplemented for 24 weeks with 5-methyltetrahydrofolate (5-MTHF, 7.5 mg /day) and vitamin B12 (0.5 mg/day). Red blood cells (RBCs) were used to investigate plasma membrane, since RBCs share lipid, protein composition and organization with other cell types. We evaluated RBCs membrane lipid composition, membrane protein oxidative damage, cation content, cation transport pathways, plasma and RBCs folate levels and plasma homocysteine levels at baseline and after 24 weeks of 5-MTHF and vitamin B12 supplementation. In CF children, 5-MTHF and vitamin B12 supplementation (i) increased plasma and RBC folate levels; (ii) decreased plasma homocysteine levels; (iii) modified RBC membrane phospholipid fatty acid composition; (iv) increased RBC K^+^ content; (v) reduced RBC membrane oxidative damage and HSP70 membrane association.

**Conclusion and Significance:**

5-MTHF and vitamin B12 supplementation might ameliorate RBC membrane features of children with CF.

**Trial Registration:**

ClinicalTrials.gov NCT00730509

## Introduction

Cystic fibrosis (CF) is one of the most common fatal autosomal recessive disorders caused by mutations of gene for the cystic fibrosis transmembrane conductance regulator (CFTR), which is a member of the transporters acting as ATP-gated chloride channel [1]–[6]. CF is a multiorgan disease mainly characterized by chronic pulmonary infections and bronchiectasia, abnormal pancreatic function, related to severe perturbation of its exocrine activities [1]–[6]. In CF patients, the most common mutation in CFTR is the deletion of a phenylalanine at position 508 (DeltaF508), which favours abnormalities in CFTR folding with fast protein degradation and loss of chloride conductance function [7]–[9].

In the last decade, the increased life expectancy of cystic fibrosis (CF) patients has significantly modified their therapeutic end-points [1]–[9]. In addition, studies on both CF animal models and CF patients suggest that besides the CFTR gene mutation, other defects such as altered activity of various membrane cation transport systems [2], [3], abnormal membrane phospholipid composition, elevated turnover of essential fatty acids (EFAs) [7], [10]–[13] and oxidant-antioxidant imbalance, can contribute to clinical manifestations of CF [14]–[17].

New experimental therapeutic strategies for CF propose a diet supplementation to affect plasma membrane fluidity and to modulate the cellular amplified inflammatory response in CF target organs. In particular, previous works have suggested a link between the CF clinical manifestations and the cell plasma membrane fluidity as supported by the correction of some pathological manifestations in supplemented CF mice [11], [17]–[19]. Recently, we have reported a case of a child with CF who has been beneficially treated with 5-methyltetrahydrofolate (5-MTHF), the active form of folic acid, since her birth. We observed amelioration of her clinical state and significant changes in red cell membrane fatty acid composition [20]. Based on these preliminary evidence, we proposed that 5-MTHF improves the red cell membrane lipid status through the increase in methionine production [21], [22]. 5-MTHF by its cofactor, vitamin B12, can deliver one-carbon unit to the methionine cycle, which is essential for methylation reactions and synthesis of DNA, RNA, proteins and phospholipids (PLs). Through this pathway phosphatidylethanolamine (PE) receives methyl groups from S-adenosyl-methionine (SAM) to form phosphatidylcholine (PC). Methylated PE induces both the increase in polyunsaturated fatty acid (PUFA) membrane concentration [23], [24] and the improvement of chloride channel activity as supported by the *in vitro* evidence in mouse L-cells expressing the deltaF508-CFTR mutated protein [24]. A defective methionine-homocysteine metabolism reduces the SAM-dependent methylation of PE, resulting in accumulation of PE and depletion of PC. An abnormally increased plasma PE /PC ratio associated with lower SAM and increased S-adenosylhomocysteine (SAH), has been recently reported in children with CF compared to normal controls, indicating a perturbation of the methylation pathway [25].

In normal adult patients, studies on 5-methyltetrahydrofolate (5-MTHF), the active form of folic acid, have shown that 5-MTHF is more effective than equimolar amount of folic acid in increasing cell folate content [26]–[28] and that 5-MTHF at high dosages has a safe profile [29]. In addition, 5-MTHF supplementation markedly increases folate plasma levels and red cells folate content, which can be used as a marker of long-term folate status, also being a correlation with liver folate concentrations [30].

Red blood cells are an interesting and experimentally easy accessible system to investigate plasma membrane, which shares lipid, protein composition and organization with other cell types. CFTR protein [16], [31]–[34] is present in human, mouse and rabbit red cells; in CF patients is present as a mutated protein. Abnormalities in red cell membrane essential fatty acid content and an increased susceptibility to oxidative damage have been recently described in CF patients, supporting the use of red cells as a cell model in CF [35]–[39]. Physiological studies on red cell membrane permeability in CF patients are contradictory and not conclusive, most likely related to the differences in the CF population studied [40]–[44].

The goal of our study was to evaluate in CF children the biological efficacy of diet supplementation with the active form of folic acid (5-MTHF) associated with vitamin B12 on red cell membrane features, used as a paradigm for other cellular plasma membranes. In children with CF we demonstrate that 5-MTHF and vitamin B12 supplementation (i) increases plasma and red cell folate levels; (ii) decreases plasma homocysteine levels; (iii) modifies red cell membrane fatty acid composition; (iv) increases red cell K^+^ content; (v) reduces red cell membrane oxidative damage; and HSP70 membrane association.

## Methods

The protocol for this trial and supporting CONSORT checklist are available as supporting information ; see Checklist S1 and Protocol S1.

### Participants

**Figure 1:**
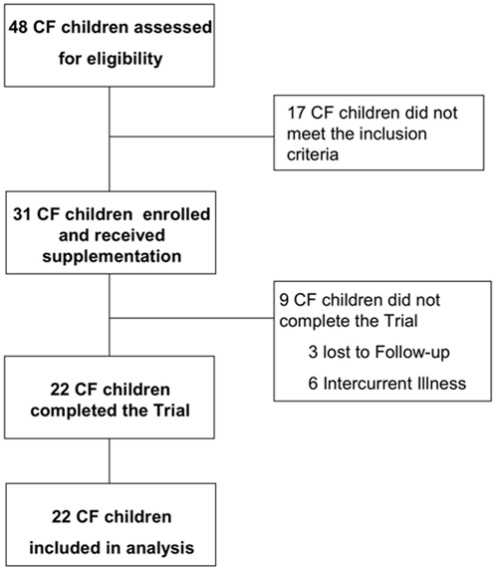
Consort style flowchart of participants through the study.

We carried out a pre-protocol study in CF children and healthy controls aged from 3 to 8 years old. We then conducted a single arm trial from April 2004 to March 2006 in a population of CF children referring to the CF Centre of Verona Hospital. Of 48 assessed CF children aged between 3 and 8 years old, thirty-one patients were enrolled in the single-arm trial (Fig. 1). Exclusion criteria were diabetes, chronic infections of the airways and regular antibiotics intake. Genotype, clinical status and medications were recorded (Table 1). Patients were monthly checked.

**Table 1:** Clinical data of cystic fibrosis patients at baseline.

Patient	Gender	Age (yr)	CFTR mutations	BMI (kg/m2)	FEV_1_ (%)	Pancreatic sufficiency	M.o. in sputum	Antibiotic treatment
**1**	M	8	DF508/DF508	17,29	81	no	no	no
**2**	F	7	DF508/DF508	21,7	101	no	*P. aeruginosa C*	azithromycin p.o.
								tobramycin neb.
**3**	F	6	DI507/711+5G A	22,5	91	no	no	no
**4**	F	5	DF508/not identified	15,2	NA	no	no	no
**5**	M	8	DF508/DF508	15,4	92	no	no	no
**6**	M	7	N1303K/2789+5G A	19,2	73	no	no	no
**7**	F	7	DF508/621+1G T	15,1	82	no	*S. aureus*	no
**8**	F	8	DF508/1717-1G T	18,3	91	no	*S. aureus*	no
**9**	F	8	DF508/not identified	21,2	95	yes	no	no
**10**	F	7	DF508/2789+5G A	14,4	93	no	no	no
**11**	M	8	DF508/2789+5G A	15,9	106	yes	no	no
**12**	F	7	R1162X/R1162X	17,15	78	no	*S. aureus*	no
**13**	M	8	DF508/not identified	15,2	63	no	no	no
**14**	M	6	DF508/DF508	17,1	115	no	*P. aeruginosa I*	ciprofloxacin p.o.
								tobramycin neb.
**15**	F	8	DF508/R1162X	14	49	no	*P. aeruginosa C*	azithromycin p.o.
								tobramycin neb.
**16**	M	5	G542/1717-1G A	16,5	NA	no	no	No
**17**	F	5	DF508/not identified	13,3	NA	no	*P. aeruginosa I*	ciprofloxacin p.o.
								tobramycin neb.
**18**	M	4	DF508/G542X	15,7	NA	no	no	no
**19**	F	7	DI507/R1162X	16,5	94	no	no	no
**20**	F	4	DF508/Q552X	13,5	NA	no	no	no
**21**	M	8	DF508/R1162X	13,8	78	no	no	no
**22**	F	6	2183AA G/N1303K	16,8	102	no	no	no

P.: Pseudomonas; S.: Staphylococcus; H.: Haemophilus; C: chronic colonization; I: intermittent colonization; NA: not applicable; p.o.: per os; neb.: nebulized.

### Ethics

The study protocol was approved by the ethic committee of the Verona Hospital and parents of children with CF provided written informed consent before starting.

### Interventions

CF children were supplemented for 24 weeks with 5-MTHF and vitamin B12 respectively at the dosage of 7.5 mg/day and 0.5 mg/day Hospital. They were asked to maintain their usual diet and the multivitamin supplementation (Protovit, Roche), throughout the study. Supplements were taken every morning before breakfast except on the blood sampling days when the supplementation was taken after venipuncture.

We evaluated RBCs membrane lipid composition, membrane protein oxidative damage, cation content, cation transport pathways, plasma and RBCs folate levels and plasma homocysteine levels at baseline and after 24 weeks of 5-MTHF and vitamin B12 supplementation.

Blood samples were collected into EDTA-coated tube and Na-heparinized tube and immediately processed. Blood samples were centrifuged and plasma was collected and stored at −80 C before analysis. Buffy-coat was removed and red cells were washed in choline washing solution (CWS) containing 172 mM choline chloride, 1 mM MgCl_2_ and 10 mM Tris-MOPS (pH 7.4 at 4°C) and used for evaluation of the following parameters: red cell cation content, cation transport pathways and red cell membrane protein profile.

### Objectives

The objective of our study was to evaluate in CF children the biological efficacy of diet supplementation with 5-MTHF and vitamin B12 on red cell membrane features.

### Determination of plasma and red cell folate content, of plasma vitamin B12, plasma homocysteine and red cell malondialdehyde levels

Both plasma and red cell folate and plasma vitamin B12 were quantified using competitive immunoassay (Diagnostic Products Corporation, USA). Plasma malondialdehyde and homocysteine were determined using high-pressure liquid chromatography (Chromsystems, Germany).

Plasma homocysteine concentrations (summation of homocysteine, homocystine and homocysteine-cysteine mixed disulphide, free and protein bound), were determined by high performance liquid chromatography (HPLC) according to a modification of the method of Araki and Sako [45], [46].

The plasma thiol compounds were reduced with tri-n-butylphosphine and derivatized with a thiol specific fluorogenic reagent: ammonium 7-fluoro-benzo-2-oxa-1,3-diazole-4-sulphonate. Subsequently, derivatives were separated by reverse phase HPLC. The fluorescence intensities were measured with excitation at 385 nm and emission at 515 nm, using a Jasko FP-2020 plus fluorescence spectrophotometer. Determination of intra- and inter-assay precision was made by quantification of the homocysteine concentration within a normal range plasma pool: the CV% values were 2.5% and 3.5% respectively. The recovery tests were >95%. The lowest validated detectable concentration of Hcy were 2 µmol/L.

Erythrocyte concentrations of MDA were determined by HPLC according to a modification of the method of Agarwal and Carbonneau [47], [48]. 1 ml of samples were adjusted to pH 13, using 50 µl of 10 mol/L NaOH and incubated in a 60°C water bath for 30 min. The hydrolyzed samples were acidified to pH 1 with 500 µl of 586 g/L perchloric acid reagent. After centrifuging at 1000×g for 10 min, 0.3 ml of the supernate was added to 50 µl of thiobarbituric acid reagent. The derivatives were separated by reverse phase HPLC. The fluorescence intensities were measured with excitation at 515 nm and emission at 553 nm, using a Jasko FP-2020 plus fluorescence spectrophotometer. Recovery tests were between 87 and 100%.

### Measurement of red cell membrane fatty acid composition

Individual phospholipids were separated by thin layer chromatography (TLC) in one dimension on 20 cm×20 cm silica plates (art. 5715, Merck, Darmstadt, Germany) which were previously washed with methanol in order to remove impurities and dried. TLC developing chamber was lined with chromatography paper on all sides. Solvents for TLC were mixed by vigorous shaking, added to the tank and allowed to equilibrate for 2 hours before the run. The samples were applied 2 cm from the bottom of the plate, as narrow bands (10 mm) with continuous application of lukewarm air. The plates were run in chloroform∶methanol∶acetic acid∶water (25∶15∶3∶1,5 v/v/v/v). The run was stopped when the solvent front reached the top and the plate was fully dried with a hair dryer (cold air). After exposure of the TLC plate to iodine vapour, each phospholipid class was scraped off and extracted with 2 ml of methanol∶benzene (1.5∶0.5 v/v) mixture. A one-step transesterification reaction was performed according to Lepage [49], in order to generate fatty acids methylesters. As previously described [50], a gas chromatographic (5890 gas chromatograph, Hewlett Packard, Palo Alto, CA, USA) method was used to separate and estimate fatty acids. Analysis was performed in duplicate on each sample, peak identification was done with commercially available reference fatty acids (Sigma) and heptadecanoic acid (C17∶0) was used as internal standard. Fatty acid results are expressed as g/100 g total fatty acids. We used unsaturation index (UI), as parameter to evaluate membrane fluidity [51]. UI was calculated by the sum of (number of double bonds in each fatty acids x % each fatty acid).

### Measurements of red cell cation content and Na/K pump, Na/K/2Cl cotransport and Na/H exchange activities in red cells

Erythrocyte Na^+^ and K^+^ content were determined by atomic absorption spectrometry (ANALYST 2000, Perkin-Elmer) using standards in double-distilled water [52]–[54]. Cation transport activities were estimated according to previously published methods [52]–[54].Briefly, the maximal rates of Na/K pump and Na/K/Cl cotransport (cot) actitvities were measured in 50 mmol L^−1^ of cells containing equal amounts of Na^+^ and K^+^. With this procedure the internal sites for both transport systems were saturated. The nystatin loading solution contained 70 mmol L^−1^NaCl, 70 mmol L^−1^ KCl and 55 mmol L^−1^ sucrose. The Na/K pump was estimated as ouabain sensitive fraction of Na efflux into a medium containing 130 mmol L^−1^ choline chloride and 10 mmol L^−1^ KCL. The ouabain concentration was 0.1 mmol L^−1^, Na/K/Cl/ COT estimated as bumetamide sensitive fraction of Na efflux into a medium containing 140 mmol L^−1^ choline chloride and 0.1 mmol L^−1^ oubain. The bumetamide concentration was 0.01 mmol L^−1^. All media contained 1 mmol L^−1^ MgCl_2_, 10 mmol L^−1^ glucose, and 10 mmol L^−1^ Tris-MOPS pH 7.4. The Na/H exchange rate was evaluated as the amiloride-sensitive Na^+^ efflux stimulated by hypertonic shrinkage from cells containing equal amounts of Na^+^ and K^+^. The media contained 140 mmol L^−1^ choline Cl and the osmolarity was increased with sucrose. The 5-N,N-hexamethyleneamiloride 10 mmol L^−1^ final concentration was used as a specific inhibitor of the system.

### Red cell membrane ghost preparation

Red cell ghosts were obtained as previously described [55]. Briefly, red cells were washed 4 times with choline washing solution (in mM CWS: 155 choline chloride, 1 MgCl_2_, 10 Tris-Mops pH 7.4 at 4°C, 295–300 mOsm). Red cell ghosts were obtained lysing 1 volume of packed red cells in 10 volumes of ice cold Phosphate Lysis Buffer (in mM PLB: 5 Na_2_HPO_4_ pH 8.0, in presence of protease inhibitor cocktail tablet, 3 benzamidine, 1 Na_3_VO_4_). Samples were incubated for 10 min in ice and centrifuged for 10 min at 12,000 g, 4°C. Ghosts were then washed four times (centrifuging at 12,000 g, 4°C) with PLB until they appeared almost white and used for either fluorescein-5-maleimide analysis or electrophoresis analysis.

Red cell membrane protein oxidative damage was evaluated using fluorescein-5-maleimide, which labels the proteins with active thiol-groups [55]. Red cell ghost were diluted with buffer (PBS) to final volume of 1 ml at 4°C. Fluorescein-5-maleimide was solubilized in PBS (0.25 mg/ml). Red cell ghost were then incubated with fluorescein-5-maleimide for 1 hour at room temperature under dark. Red cell ghost were washed 5 times with PBS, then were mixed with sample buffer and separated by mono- dimensional electrophoresis (GE Healthcare). Fluorescence was detected by placing the gel on a UV light source, photographed and analyzed. The bands showing different fluorescence were excised in the corresponding Colloidal Coomassie stained gels and used for protein identification by MALDI-TOF [56], [57].

### MALDI-TOF MS analysis and database search

The bands from 1D gels differently expressed in Colloidal Coomassie stained gels were excised, destained (in DS: 50% Acetonitrile, 5 mM NH_4_HCO_3_), dehydrated in 100% acetonitrile and digested overnight at 37°C with a trypsin solution (0.01 mg/ml trypsin, 5 mM NH_4_CO_3_). Mass spectra analysis was performed using a Tof-spec SE (Micromass, Manchester, UK) or MALDI-Micro MX mass spectrometer with PSD technology (Micromass, Manchester, UK). Peptide spectra were obtained in positive ion mode over the m/z range of 800–4000 Da range or 1000–3000 Da in reflectron mode. Peptide solution was prepared mixing equal volumes of matrix (matrix: α-cyano-4-hydroxy-cynnamic acid 8 mg in 40% acetonitrile, 60% of 0.1% trifluoracetic acid). 100–120 laser shots were summed for each MS spectrum. Database searching was performed using the measured peptide masses against the Swiss-Prot database (*taxa human*) using the MASCOT search engine (Matrix Science Ltd, London, UK) Only protein identifications with significant Mascot score (p<0.05) were taken in consideration. A mass accuracy of 0.3 Da and a single missed cleavage were allowed for each matching peptide. Searches were not constrained by pI or molecular weight [56], [57].

### Immunoblot analysis for methylterahydrofolate reductase (MTHFR) and heat Shock Protein-70 (HSP70) in red cells

Red cell membrane ghost were solubilized and separated by mono-dimensional electrophoresis, transferred to membrane and probed with specific antibodies: anti-Methylenetetrahydrofolate reductase (MTHFR, clone N-20) antibody was from Santa Cruz Biotechnology (Santa Cruz, CA, USA); anti- heat shock protein 70 (HSP70, clone K-20) antibody was from Santa Cruz Biotechnology (Santa Cruz, CA, USA).

### Statistical methods

Wilcoxon rank-sum test was used to compare differences between healthy children and CF children at baseline in plasma and red cell folates, plasma vitamin B12, plasma homocysteine, red blood cell MDA, membrane ion pathways, red cell cation content and fatty acid percentages bound either to phosphatidylethanolamine (PE) or to phosphatidylcholine (PC).

Wilcoxon signed-rank test was used to compare differences between CF children at baseline and after 5-MTHF and vitamin B12 supplementation in fatty acid percentages bound either to PE or to PC.

## Results

### Pre-protocol study: *cystic fibrosis patients show increased red cell folate content and abnormal red cell features*

In the pre-protocol study, we evaluated the baseline characteristics of 10 healthy controls and 22 children with CF who participated at the trial and (Table 2). In CF patients, plasma folate levels were similar to healthy children while red cell folate content was significantly higher. Plasma vitamin B12 concentration was increased in children with CF compared to healthy subjects, most likely related to the presence of this vitamin in low quantity in multivitamin tablets assumed by CF children before the study. Plasma homocysteine levels were similar in CF patients and healthy children, while previous studies reported an increase in plasma homocysteine levels in CF subjects. This discrepancy might be related to diet differences between populations.

**Table 2:** Data of healthy subjects and cystic fibrosis (CF) patients.

	Controls	CF patients	p-value
**Age** (years)	5.9±2.1	6.7±1.4	
**Sex** (M/F)	5/5	9/13	
**MTHFR 677 C>T polymorphism**	NA	w/w 42 %	
	NA	w/m 35 %	
	NA	m/m 23 %	
**Plasma folate** (ng/ml)	5.88 (4.07–6.74)	6.62 (4.99–8.16)	0.174
**RBCs folate** (ng/ml)	49.4 (27.03–82.8)	451.5 (339.0–708.0)	0.004
**Plasma vitamin B12** (pg/ml)	896 (660–1055)	1157 (871–1448)	0.159
**Plasma homocysteine** (µmol/l)	6.90 (6.70–7.90)	7.50 (6.85–8.50)	0.424
**RBCs MDA** (µmol/g Hb)	12.65 (11.45–13.85)	13.70 (11.90–16.30)	0.232

RBCs: red blood cells; N: rare mutation; NA: not applicable; MTHFR: methylenetetrahydrofolate reductase; w/w: wild; w/m: heterozygote; m/m: mutant; MDA: malondialdehyde. Data are summarized as mean±sd for age, frequency for sex and genotype, medians and interquantile ranges for folate, vitamin B12, homocysteine and MDA.

**Figure 2:**
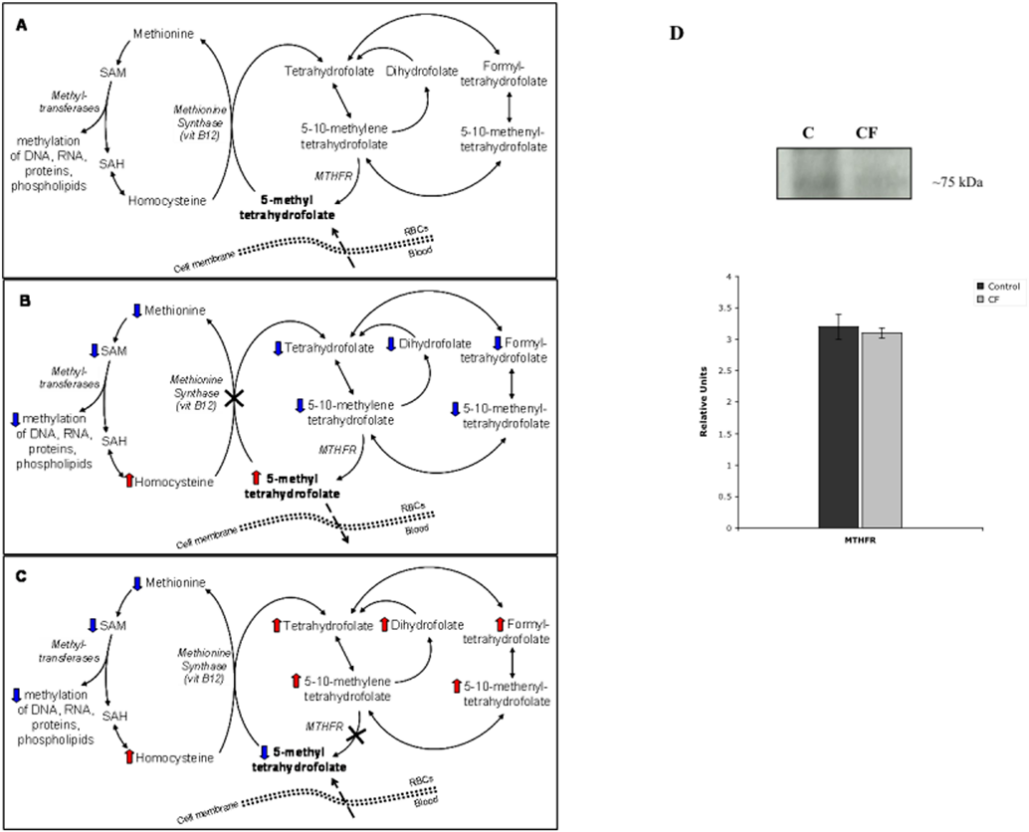
The intracellular folate metabolism. (A) Human cells receive exogenous 5-methyltetrahydrofolate (5-MTHF) from the bloodstream and produce endogenous 5-MTHF through the irreversible methylenetetrahydrofolate reductase (MTHFR) reaction that catalyzes the reduction of 5,10-methylenetetrahydrofolate to 5-methyltetrahydrofolate. 5-MTHF serves as methyl donor in the remethylation of homocysteine to methionine, which in turn is converted to S-adenosylmethionine (SAM). The transmethylation reaction, which requires vitamin B12 and the methionine synthase enzyme, converted 5-MTHF in tetrahydrofolate (THF). As 5-MTHF is a poor substrate for folylpolyglutamate synthetase, this enzyme adds glutamyl residues to non-methylated folates, guaranteeing the intracellular folate retention. (B) A reduced activity of the methionine synthase activity or a deficit of vitamin B12 causes a reduction of the intracellular folates. In fact, 5-MTHF leaks out of cells because cells cannot accumulate it, being monoglutamised and therefore shorter than other intracellular folates. Conversely, (C) a reduced activity of the methylenetetrahydrofolate reductase (MTHFR) causes the non-methylated folate accumulation, because these folates are polyglutamised by folylpolyglutamate synthase. (D) Immunoblot analysis of methythertrahydrofolate reductase (MTHFR) protein expression in red cells from healthy controls and in CF patients. One representative gel of other 20 with similar results. Graph reporting the immuno-blot analysis for the quantification of MTHFR expression as detected by densitometry in normal controls (black bar) and in CF patients (gray bar); data are presented as percentage of baseline values (*n* = 20).

The increase in red cell folate content was unexpected, suggesting that folates may accumulate as non-active form in red cells of CF patients. Previous reports in other cell types indicate that intracellular folates can be present as active form, or 5-methyltetrahydrofolate (5-MTHF), and non-active forms as polyglutamated folates [58]–[60]. While non-methylated folates cannot cross the plasma membrane and might function as cellular reservoir, 5-MTHF can easily cross the cell membrane. The intracellular production of 5-MTHF is dependent on methylenetrahydrofolate reductase (MTHFR) activity (Fig. 2A) [58]. Thus, we hypothesized that in CF patients the intracellular non-methylated folates are accumulated, while the 5-MTHF is not produced, most likely due to alterations of MTHFR activity (Fig. 2C), as also supported by the normality of vitamin B12 cell content, whose deficiency can contribute to folate trapping in cells (Fig. 2B) [59], [60]. In order to validate this hypothesis, we evaluated the incidence of C677T methylenetrahydrofolate reductase (MTHFR) polymorphisms in the studied CF patients and no differences were observed compared CF to historical controls of local population (Table 2) [61]. We then evaluated the protein expression of MTHFR in red cells from normal and CF children. As shown in Fig. 2D, MTHFR protein expression was similar in normal and CF red cells, suggesting that the alteration of 5-MTHFR function might be most likely responsible for the increased red cell folate content in CF patients.

Abnormalities of MTHFR function, determining reduced biological availability of 5-MTHF, might promote alteration of membrane lipid status and abnormal oxidative scavenging [21]–[23]. In fact, 5-MTHF increases the methionine production, which promotes the phosphatidylethanolamine (PE) methylation with increased binding of polyunsaturated fatty acids (PUFAs) [23]. Thus, we evaluated membrane phospholipid fatty acid composition of red cells and monocytes from CF patients. As shown in Table 3, polyunsaturated fatty acids (PUFAs) were lower in red cell phospholipids of CF subjects than in healthy controls, confirming a previous report [25] and suggesting a possible perturbation in fatty acid metabolism [24], [25]. Similar data were also observed in CF monocytes (data not shown). The unsaturation index (UI), that is the sum of all double bonds in fatty acid chains, was slightly reduced in both red cells and monocytes from CF patients compared to healthy children (CF RBCs UI: 144.9±10.1 *vs* normal RBCs: 145.81±2.31; CF- monocyte UI: 133.5±13.07 *vs* normal 134.4±4.21), but it did not reach significance.

**Table 3:** Comparison of red cell phospholipid fatty acid composition in healthy subjects and patients with cystic fibrosis (CF).

	Phospholipid fatty acids	Controls	CF patients	p-value
**PE**	**SFA**	61,1 (60,6–61,8)	67,8 (52,2–69,0)	0,317
	**MUFA**	18,6 (18,2–19,8)	27,2 (26,0–33,5)	0,019
	**PUFA**	19,6 (18,2–20,7)	5,1 (3,4–9,9)	0,002
	**UFA**	38,9 (38,2–39,4)	33,2 (31,0–47,8)	0,317
	**W3**	1,1 (1,1–1,4)	0,5 (0,4–0,6)	0,019
	**W6**	18,2 (17,1–19,3)	4,6 (3,1–8,4)	0,002
**PC**	**SFA**	60,7 (58,1–60,8)	74,3 (67,5–78,1)	0,002
	**MUFA**	24,1 (23,8–24,5)	18,4 (16,6–24,3)	0,125
	**PUFA**	16,2 (15,3–19,5)	7,6 (4,0–8,3)	0,002
	**UFA**	39,3 (39,2–41,9)	25,7 (21,9–32,5)	0,002
	**W3**	<0,01 (<0,01–<0,01)	0,4 (0,3–0,5)	0,002
	**W6**	16,2 (15,3–19,5)	7,3 (3,5–8,1)	0,002

PE: phosphatidylethanolamine; PC: phosphatidylcholine; SFA: saturated fatty acids, MUFA: monounsaturated fatty acids, PUFA: polyunsaturated fatty acids; UFA: unsaturated fatty acids; W3: n-3 fatty acids; W6: n-6 fatty acids. Data are summarized as medians and interquantile ranges.

**Figure 3:**
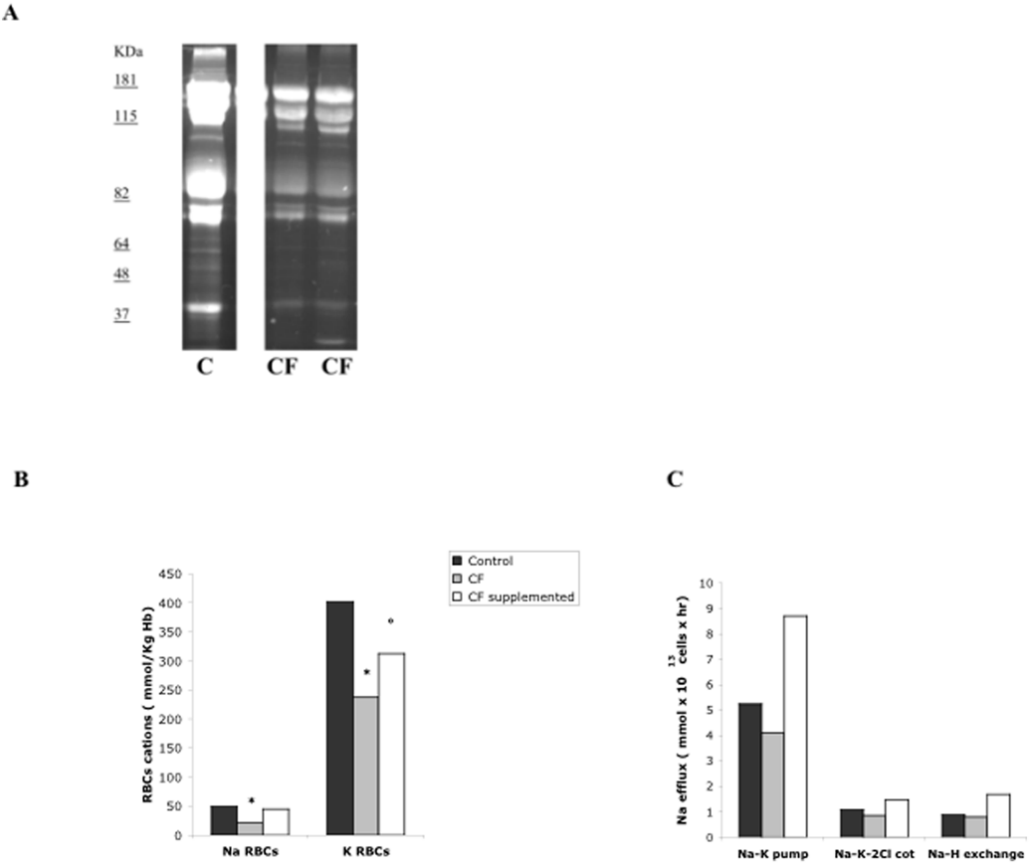
Membrane proteins, ion content and cation transport pathways of red blood cells from healthy controls and CF patients. (A) Red cell membrane proteins were separated by mono-dimensional electrophoresis and labelled with fluorescein-5-maleimide, which labels the proteins with active thiol-groups. Fluorescence was detected by placing the gel on a UV light source, photographed and analyzed. One representative gel of other 10 with similar results. (B) Red cell Na^+^ and K^+^ content in healthy controls and CF patients at baseline and after 5-MTHF and vitamin B12 supplementation for 24 weeks. Data are reported as medians (range for controls: RBCs Na^+^ 28.4–74.3 mmol/Kg Hb, RBCs K^+^ 320–512 mmol/Kg Hb *n* = 10; CF at baseline: RBCs Na^+^ 19.2–41.6 mmol/Kg Hb, RBCs K^+^ 199.7–335 mmol/Kg Hb *n* = 20; CF supplemented with 5-MTHF and vitamin B12: RBCs Na^+^ 15.6–61 mmol/Kg Hb, RBCs K^+^ 236–447 mmol/Kg Hb, *n* = 11); * *P*<0.05 compared to control red cells; ° *P*<0.05 compared to untreated CF patient red cells. (C) Cation transport pathways of red cells from controls (*n* = 10) and CF patients at baseline (*n* = 11) and after supplementation (*n* = 11). Data are expressed as medians (range for controls: Na-K pump 4.1–6.2 mmol×10^13^ cells×hour, Na-K-2Cl cotransport 0.98–1.90 mmol×10^13^ cells×hour, Na-H exchange 0.68–1.2 mmol×10^13^ cells×hour; CF baseline: Na-K pump 3.28–11 mmol×10^13^ cells×hour, Na-K-2Cl cotrasnport 0.15–1.70 mmol×10^13^ cells×hour, Na-H exchange 0.21–1.20; CF supplemented: Na-K pump 3.70–10.0 mmol×10^13^ cells×hour; Na-K-2Cl cotrasnport 0.49–4.70 mmol×10^13^ cells×hour; Na-H exchange 1.0–5.0 mmol×10^13^ cells×hour); * *P*<0.05 compared to control red cells; ° *P*<0.05 compared to untreated CF patient red cells.

Previous reports have suggested that 5-MTHF exerts antioxidant properties as scavenger of peroxynitrite [62]. Thus, the following parameters of oxidative damage we evaluated: red cell levels of malondialdehyde (MDA) as lipid peroxidation end product [63] and the membrane protein binding of fluorescein-5-maleimide, which labels proteins at level of active thiol-groups, as marker of membrane protein oxidative damage [55]. Red cell MDA content was slightly but not significantly higher in CF patients compared to normal subjects (Table 2). As shown in Fig. 3A, we observed reduced fluorescence intensity of red cell membrane proteins in CF patients compared to healthy subjects, suggesting increased membrane protein oxidative damage in red cells from CF patients.

Since increased oxidative damage might also affect red cell cation content and the activity of membrane cation transport pathways, we evaluated the red cell Na^+^ and K^+^ content and the activities of the main cation transport pathways in CF children and in normal controls. In CF patients, Na^+^ and K^+^ content was significantly lower than in normal controls (Fig 3B), while no significant differences were observed in the activity of main membrane cation transport systems (Fig. 3C). Interestingly, the activity of the Na/K pump was slightly but not significantly decreased in CF patients compared to healthy children (Fig. 3C).

Baseline data suggest reduced membrane fluidity and increased pro-oxidant environment in CF red cells, affecting both lipid composition and protein components of red cell membrane. Thus, we investigated whether dietary supplementation with 5-MTHF, the active form of folic acid, and vitamin B12, might by-pass the hypothesized functional block of MTHFR and ameliorate red cell features.

### Single arm trial: *supplementation with the active form of folic acid and vitamin B12 in children with CF ameliorates red cell membrane features and reduces heat shock protein 70 membrane association*

Thirty-one patients were enrolled in the single-arm trial. Three patients withdrew from the study due to loss to follow-up and six patients for intercurrent illness. All the other patients completed the trial (Fig. 1). No difference in baseline measurements had been documented between the 9 patients who dropped out the study and the other 22 who completed the trial. No adverse events were registered during the trial.

**Figure 4:**
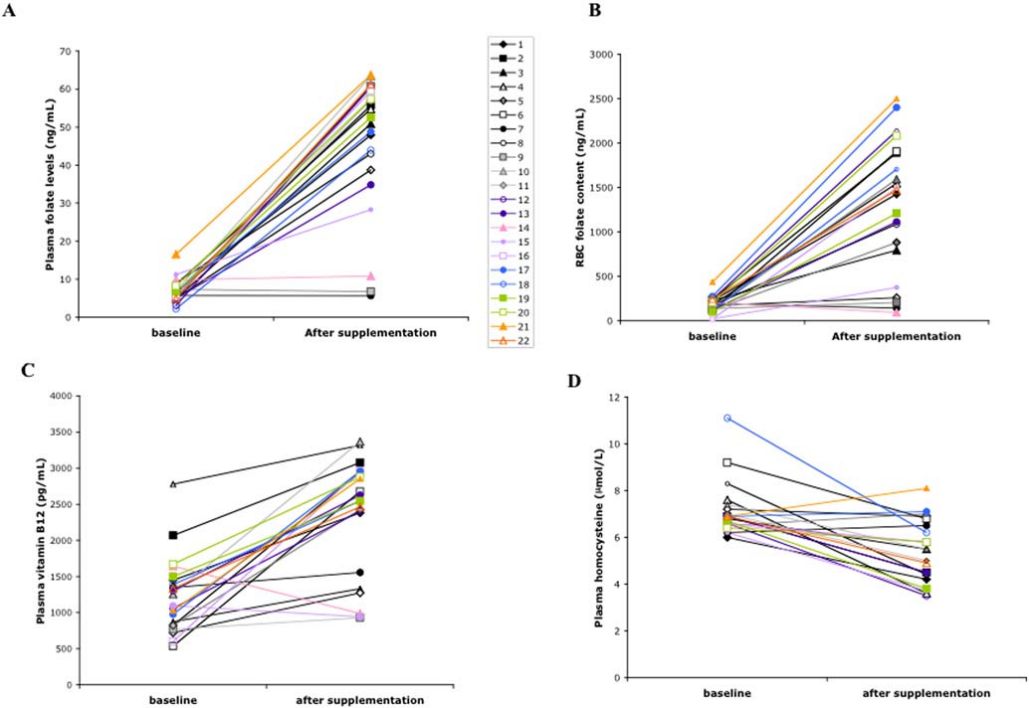
Effects of 5MTHF and vitamin B12 supplementation on levels of plasma and red cell folate, of plasma vitamin B12 and homocysteine in CF patients. (A) Plasma folate levels were 6.62 ng/mL (range: 4.99–8.16 ng/mL) at baseline and 52.85 ng/mL (range: 47.95–60.51 ng/mL) after supplementation; (B) red cell folate levels were 451.50 ng/mL (range: 339.00–708.00 ng/mL) at baseline and 4800.27 ng/mL (range: 3624–6249 ng/mL) after supplementation; (C) plasma vitamin B12 levels were 1157 pg/mL (range: 871–1448 pg/mL) at baseline and 2697.09 pg/mL (range: 2463–2963 pg/mL), after supplementation; (D) plasma homocysteine levels were 7.50 µM/L (range: 6.85–8.50 µM/L) at baseline and 5.30 µM/L (range: 4.42–6.21 µM/L) after supplementation.

Supplementation with 5-MTHF and vitamin B12 significantly increased plasma folate levels, red cell folate content and plasma vitamin B12 levels and significantly reduced plasma homocysteine levels in children with CF (Fig. 4). However, some of the CF patients (*n* = 6) did not show a total compliance to the 5-MTHF and vitamin B12 supplementation as supported by the unmodified values of red cell folate content, which may be considered a marker of long-term (24 weeks) patient folate state [27], [28].

At the end of the study we observed a significant increase of PUFAs bound to PE in red cell membranes (Table 4, *P* = 0,038) as well as in CF monocytes (data not shown) indicating that 5-MTHF and vitamin B12 supplementation beneficially modified the fatty acid composition of red cell membrane phospholipids. An increased incorporation of n-6 fatty acid accounted for the higher proportion of PUFAs in PE after the supplementation (Table 4). In fact, arachidonic acid (AA) bound to PE both in red cell and in monocyte membrane was significantly increased (from 10.31±1.82 to 15.93±2.80, respectively, in red cells), suggesting that a lower amount of AA may be released for generating eicosanoids, which participate in inflammatory injury and have been reported to be increased in CF patients [64]. In CF patients supplemented with 5-MTHF and vitamin B12, the unsaturation index (UI) both in red cells and in monocytes was significantly increased, indicating an improvement of membrane fluidity (RBCs baseline UI: 144.9±10.1 *vs* treated: 147.64±9.9, *P*<0.05; monocytes baseline UI: 133.5±13.07 *vs* treated: 140.7±11.63, *P* = 0.06). Interestingly no significant difference in plasma UI was present in CF patients before and after supplementation (baseline UI 122.11±2.3 *vs* treated: 122.72±2.61; *P* = ns).

**Table 4:** Effects of 5-methyltetrahydrofolate and vitamin b12 supplementation on red cell phospholipid fatty acid composition of patients with cystic fibrosis (CF).

	Phospholipid fatty acids	CF baseline	CF after supplementation	p-value
**PE**	**SFA**	67,8 (52,2–69,0)	66,6 (52,0–67,0)	0,138
	**MUFA**	27,2 (26,0–33,5)	27,7 (27,0–33,0)	0,952
	**PUFA**	5,1 (3,4–9,9)	5,8 (5,2–12,4)	0,038
	**UFA**	33,2 (31,0–47,8)	33,4 (33,0–48,0)	0,26
	**W3**	0,5 (0,4–0,6)	0,6 (0,4–0,7)	0,514
	**W6**	4,6 (3,1–8,4)	5,1 (4,8–10,9)	0,028
**PC**	**SFA**	74,3 (67,5–78,1)	76,7 (68,9–78,5)	0,161
	**MUFA**	18,4 (16,6–24,3)	18,1 (15,5–23,4)	0,161
	**PUFA**	7,6 (4,0–8,3)	7,3 (3,8–8,2)	0,123
	**UFA**	25,7 (21,9–32,5)	23,3 (21,5–31,1)	0,161
	**W3**	0,4 (0,3–0,5)	0,3 (0,3–0,4)	0,182
	**W6**	7,3 (3,5–8,1)	7,1 (3,4–7,9)	0,161

PE: phosphatidylethanolamine; PC: phosphatidylcholine; SFA: saturated fatty acids, MUFA: monounsaturated fatty acids, PUFA: polyunsaturated fatty acids; UFA: unsaturated fatty acids; W3: n-3 fatty acids; W6: n-6 fatty acids. Data are summarized as medians and interquantile ranger.

**Figure 5:**
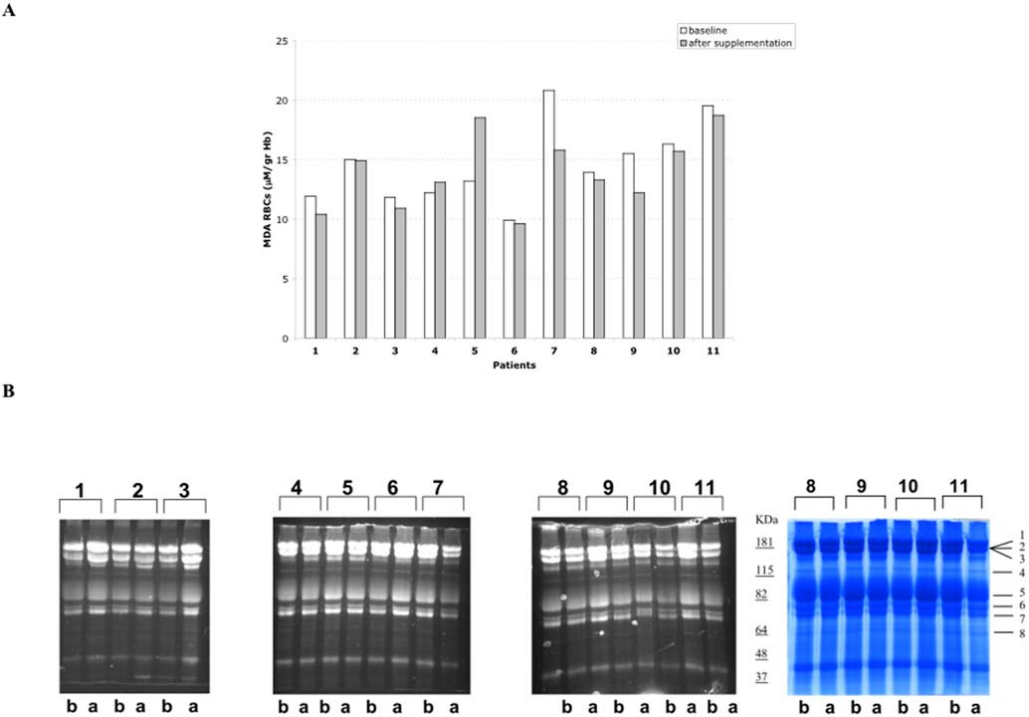
Effects of 5-MTHF and vitamin B12 supplementation on malondialdehyde content and membrane proteins of red blood cells from CF patients. (A) Malondialdehyde content of red blood cells at baseline and after supplementation (B) Red cell membrane proteins were separated by mono-dimensional electrophoresis and labelled with fluorescein-5-maleimide, which labels the proteins with active thiol-groups. Fluorescence was detected by placing the gel on a UV light source, photographed and analyzed. CF patients are indicated by numbers from 1 to 11; b: baseline; a: after supplementation with 5-MTHF and vitamin B12. The bands showing different fluorescence are indicated on the colloidal Commassie stained gel with numbers from 1 to 8.

In order to evaluate the biological effects of 5-MTHF and vitamin B12 supplementation, we further studied a group of 11 patients, 8 compliant and 3 non-compliant, and we also considered their clinical conditions and pharmacological treatments (Table 5). 5-MTHF and vitamin B12 supplementation induced a significant decrease in MDA red cell content in CF patients fully compliant with supplementation (patients: 1, 3, 6, 7, 8, 9, 10, 11, Fig. 5A), suggesting an amelioration of membrane lipid peroxidation, while in the non-compliant (patients: 2, 4, 5) we observed either no changes or a slight increase in red cell MDA levels. In the same CF patients, red cell membrane protein oxidative damage was evaluated by fluorescein-5-maleimide, which labels proteins with active thiol-groups. As shown in Fig. 5B, 5-MTHF and vitamin B12 supplementation markedly increased the intensity of fluorescence in compliant patients, suggesting a reduction of the oxidative stress in red cell membrane proteins. Patients 2, 7, 8, 10 and 11 were colonized by micro-organisms and under antibiotic treatment at the end of the trial (Table 5). These patients showed no or minor positive changes in the parameters of the oxidative damaged measured.

**Table 5:** Clinical data of subset cystic fibrosis patients.

Patient	M.o. in sputum at baseline	Antibiotic treatment at baseline	M.o. in sputum at the end of supplementation	Antibiotic treatment at the end of supplementation
**1**	no	no	no	no
**2**	*P. aeruginosa C*	azithromycin p.o.	*P. aeruginosa C*	azithromycin p.o.
		tobramycin neb.		tobramycin neb.
**3**	no	no	no	no
**4**	no	no	no	no
**5**	no	no	no	no
**6**	no	no	no	no
**7**	*S. aureus*	no	*P. aeruginosa I*	ciprofloxacin p.o.
				tobramycin neb.
**8**	*S. aureus*	no	*P. aeruginosa I*	ceftazidime i.v.
				tobramycin neb.
**9**	no	no	no	no
**10**	no	no	*H. influenzae*	cotrimoxazole p.o.
**11**	no	no	*P. aeruginosa I*	ciprofloxacin p.o.

P.: Pseudomonas; S.: Staphylococcus; H.: Haemophilus; C: chronic colonization; I: intermittent colonization; NA: not applicable; p.o.: per os; neb.: nebulized; i.v.: intravenous.

In order to identify the proteins affected by oxidative damage in CF RBCs the bands showing changes in fluorescence intensity were excised and analysed by mass spectrometry. We identified three groups of proteins, clustered according to their functions: (i) membrane proteins, (ii) cytoskeletal proteins and (iii) chaperones, suggesting that in CF patients the increase oxidative damage affects the organization and the stability of plasma membrane proteins (Table 6).

**Table 6:** List of identified proteins displaying different degrees of oxidation in red cells from normal and cystic fibrosis patients.

Band	AC	Protein Description	Matching peptides	Coverage (%)
**1**	P02549	Alfa spectrin	28	43
**2**	P11277	Beta spectrin	26	40
**3**	P16157	Ankyrin	20	22
**4**	P02730	Band 3	10	33
**5**	P11171	Band 4.1	11	26
**6**	P11171	Band 4.1	10	24
**7**	P16452	Band 4.2	9	12
**8**	P08107	Heat Shock Protein 70 (HSP70)	16	31

The corresponding bands are indicated in Fig. 4B; AC: accession number.

**Figure 6:**
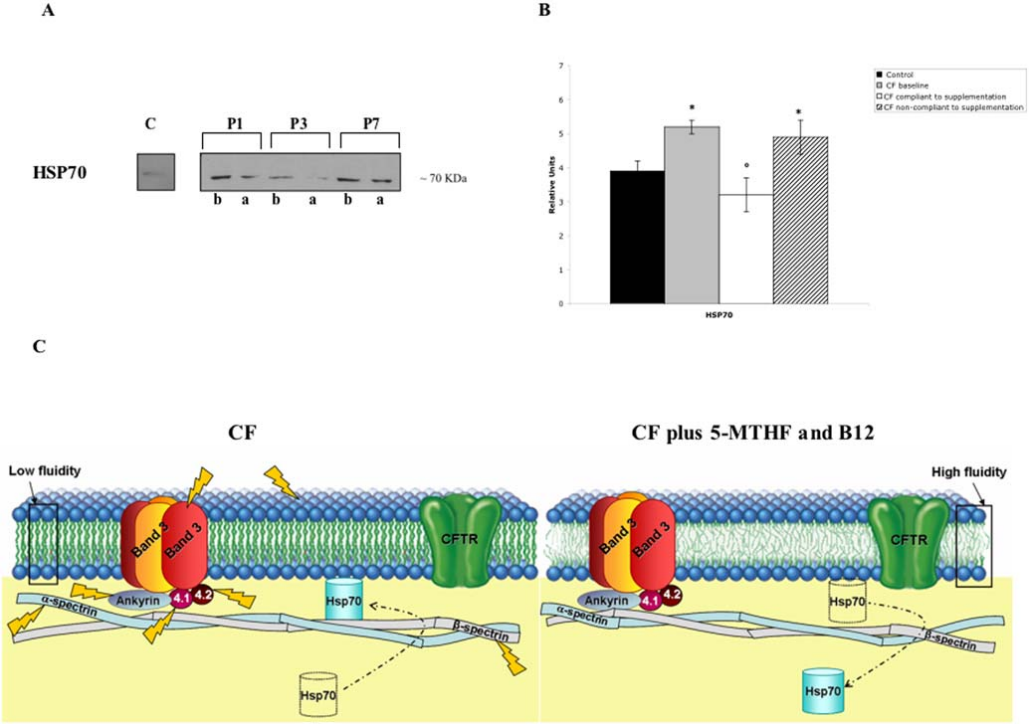
Effects of 5MTHF and vitamin B12 supplementation on heat shock protein 70 associated with red cell membrane from CF patients. (A) HSP70 associated with red cell membrane from CF patients were separated by mono-dimensional electrophoresis. Normal red cells are in line 1; red cells respectively from patient 1 (P1), patient 3 (P3) and patient 7 (P7) are in lanes 2, 4, 6 at baseline and in lanes 3, 5, 7 after treatment. (B) Graph reporting the immuno-blot analysis for the quantification of HSP70 expression as detected by densitometry in normal controls (black bar) and in CF patients (gray bar); data are presented as percentage of baseline values (*n* = 11). (C) Schematic diagram of the working hypothesis. Cellular membrane lipids and proteins are exposed to chronic oxidative stress in CF patients. The altered metabolism of polyunsaturated fatty acids (PUFAs) contributes to cellular damage, reducing membrane fluidity and increasing pro-oxidant environment. The 5-MTHF and vitamin B12 supplementation increases the levels of PUFAs and reduces both protein oxidative damage and HSP70 association on red cell membranes of CF patients.

Chaperones are group proteins (i.e. heat shock proteins) that assist and protect intracellular proteins during cell life to maintain their functional conformation [65]. In CF patients heat shock proteins have been reported to play a crucial role in the CFTR protein folding but also in the impaired CFTR chloride conductance [66]. Here, the amount of membrane associated heat shock protein 70 (HSP70) was higher in CF patients than in controls, suggesting the presence of membrane damage in CF red cells. After 5-MTHF and vitamin B12 supplementation, we observed a marked reduction in HSP70 membrane association in totally compliant CF patients compared to non-compliant, indicating a reduction in red cell oxidative stress (Fig. 6).

5-MTHF and vitamin B12 supplementation also increased red cell K^+^ content with a slight but not significant increase in red cell Na^+^ content (Fig. 3). The activity of Na/K pump, Na/K/Cl cotransport and Na/H exchange was not modified by 5-MTHF and vitamin B12 supplementation (Fig. 3C).

## Discussion

In the present study, we observed that CF children had red cell folate concentrations higher than healthy controls with associated abnormalities of red cell membranes, suggesting that the intracellular folates might be metabolically inactive. Three different mechanisms might be involved: (i) the inhibition of the phosphatidylethanolamine N-methyltransferase; (ii) the downregulation of the methionine cycle or (iii) the reduced methylenetetrahydrofolate reductase (MTHFR) activity. We exclude an inhibition of the phosphatidylethanolamine N-methyltransferase because the activity of this enzyme has been reported to be normal in CF patients [67]. A down-regulation of the methionine synthase is not plausible because, if this were the case, we would observe a reduction of red cell folate content compared to normal controls (Fig 2B) [59]. Thus, the remaining hypothesis is a reduced activity of the MTHFR associated with an accumulation of folates [60] in red cells, as we observed in erythrocytes of children with CF (Fig. 2C). Since we did not detect differences either in the incidence of MTHFR polymorphisms or in the amount of MTHFR protein in CF patients compared to normal subjects, we suggest that posttranslational modifications of the MTHFR, such as phosphorylation [68], might favour the inactive state of MTHFR in CF patients. In fact, supplementation with 5-MTHF, the active form of folate, induced a further increase in red cell folate content and a significant reduction in plasma homocysteine levels with amelioration of plasma membrane features in CF red cells and monocytes.

When 5-MTHF content was elevated by diet supplementation in CF erythrocytes, we observed an increased amount of polyunsaturated fatty acids (PUFAs), in particular of arachidonic acid (AA, n-6 fatty acid), in CF red cell plasmamembrane associated with increased unsaturation index. It is interesting to note that an isolated case of a CF child treated since her birth with active folic acid, showed increased docosahexaenoic acid (DHA, n-3 fatty acid), which was not detectable in the present study [20]. Since AA and DHA compete for the same site of esterification on phospholipids, the different diet of the children studied should be taken into account. In fact, the bioavailability of PUFAs differs between breast milk, where DHA is more abundant, and the diet of preschool-school children, characterized by n-6 fatty acids predominance [69]. However, both these studies show increased amount of unsaturated fatty acids in red cell membranes, which has been reported to improve cellular membrane fluidity. The increased levels of n-6 fatty acids, that we observed at the end of this study, also suggests a reduced release of AA from phospholipids to form eicosanoids that may contribute to inflammation in CF patients [70], [71].

Previous reports have shown increased susceptibility of CF red cells to oxidative injury [36], [38], [39], [72]–[74]. Here, we have chosen to evaluate both lipid and protein oxidative damage at baseline and after treatment. In CF red cells, we showed an increased oxidative damage of membrane proteins at baseline, suggesting that the lower availability of 5-MTHF and the accumulation of non-methylated folates might amplify the cellular pro-oxidant environment, being 5-MTHF a natural antioxidant scavenger for peroxynitrite [42]. 5-MTHF and vitamin B12 supplementation increased the bioavailability of active form of folic acid and reduced free radical cell injury as supported by the reduction in MDA red cell content and the positive changes in red cell membrane proteins of CF children (Fig. 5). In addition, 5-MTHF and vitamin B12 supplementation normalized CF red cell Na^+^ content and increased red cell K^+^ content towards values similar to those observed in normal controls, indicating an amelioration of membrane trafficking. The beneficial effect of 5-MTHF and vitamin B12 supplementation on red cell membrane is also supported by the reduction in the amount of membrane associated heat shock protein 70 (HSP70) (Fig. 6C).

Taking into account the limitations resulting from the lack of a control group who did not receive the vitamin supplementation, we can only suppose that 5-MTHF and vitamin B12 treatment might represent a new tool to ameliorate plasma membrane features in CF children. Further studies need to be carried out in order to evaluate the effective effects of 5-MTHF and vitamin B12 supplementation in a large population of children with CF.

## Supporting Information

Protocol S1 Trial protocol
(0.05 MB DOC)

Checklist S1 CONSORT checklist
(0.06 MB DOC)
